# Characterization and Quantitative Analysis of Crack Precursor Size for Rubber Composites

**DOI:** 10.3390/ma12203442

**Published:** 2019-10-21

**Authors:** Hao Guo, Fanzhu Li, Shipeng Wen, Haibo Yang, Liqun Zhang

**Affiliations:** 1 State Key Laboratory of Organic-Inorganic Composites, Beijing University of Chemical Technology, Beijing 100029, Chinazhanglq@mail.buct.edu.cn (L.Z.); 2 Key Laboratory of Beijing City on Preparation and Processing of Novel Polymer Materials, Beijing University of Chemical Technology, Beijing 100029, China; wensp@mail.buct.edu.cn (S.W.); yanghb@mail.buct.edu.cn (H.Y.)

**Keywords:** rubber composite, crack precursor, tear energy, fatigue crack growth rate

## Abstract

In the field of engineering, the annual economic loss caused by material fatigue failure reaches 4% of the total economic output. The deep understanding of rubber fatigue failure can help develop and prepare rubber composites with high durability. The crack precursor sizes within the rubber composites are vital for the material mechanical and fatigue properties. In this study, we adopted three different characterization methods to analyze crack precursor sizes and their distribution. First, based on the theoretical formula of fracture mechanics, the size of the crack precursor was deduced from 180 μm to 500 μm by the uniaxial tensile experiment combined with tear test (nicked angle tear, planar tear and trouser tear). Second, by combining the uniaxial fatigue test of dumbbell specimen with the fatigue crack growth rate test, the average size of the crack precursor was calculated as 3.3 μm based on the Thomas fatigue crack growth model. Third, the average size of the crack precursor was 3.6 μm obtained by scanning electron microscope. Through theoretical calculations and experimental tests, the size and distribution of the crack precursors of rubber composites were systematically presented. This work can provide theoretical guidance for the improvement of fatigue performance of rubber composites.

## 1. Introduction

Rubber composites are extensively used in the manufacture of vibration isolators, elastic bearings, tires, seals, gaskets, and other rubber products due to its good elastic, mechanical, and dynamic properties [1,2]. However, the rubber composites materials will gradually break and fail due to the initiation and growth of cracks in the long-term dynamic processes. The failure of rubber composites will bring great safety risks and economic losses to society. For example, when silicone rubber prosthetic materials first entered the market in 1962, there was no in-depth study on the fatigue performance of the material, which eventually led to many fatigue failure cases. The companies that launched this product (Corning, Baxter, 3M, etc.) were fined $3.4 billion [3]. So, it is imperative for us to put great importance on rubber fatigue. Fatigue is a very complicated dynamic process [4] in which the inherent defects in the materials gradually develop into cracks under cyclic stress and eventually lead to local fracture [5,6,7].

Generally speaking, the fatigue failure of rubber composites is divided into two stages: Crack nucleation stage, the crack precursors inside the rubber composites gradually grow into small cracks under the dynamic stress. And crack propagation stage, the small cracks expand further under the dynamic stress until the rubber composites break down. Corresponding to different stages, current research methods for rubber fatigue include crack nucleation method [8,9,10,11,12,13] and crack propagation method [14,15,16,17], which are often used to predict the fatigue life of rubber [18,19,20,21].

During the fatigue test, the number of cycles in the crack nucleation stage is more important than the crack propagation stage. This is because, for specific component geometry and load level, the fatigue life of the crack nucleation stage accounts for more than 90% of the total fatigue life of the rubber composite [22]. What is more worth noting is that the crack nucleation stage always occurs before the component fails, but the crack propagation may not occur (because the small initial crack may be enough to cause the component to fail due to the stiffness drop). Therefore, prevention of crack nucleation is often regarded as the main design goal.

Le Cam et al. [23,24] observed the crack propagation process of the rubber composites by in situ scanning electron microscopy and found that the original internal defects were formed by the interface slip between zinc oxide and the rubber matrix. Under cyclic dynamic stress, these defects usually became the initiation points of crack nucleation. These original defects were crack precursors, whose sizes (*c*_0_) were usually ranged from 0.01 mm to 0.1 mm [22]. Although the sizes of the crack precursors were small, they were fatal for rubber composites. During the fatigue process, after many cycles, the crack precursors inside the rubber will gradually grow to visible small cracks, which would eventually lead to the failure of the rubber composites and bring unpredictable dangers.

Besides, in rubber composites, carbon black (CB) particles tend to form CB agglomerates in processing. The appearance of stress concentration and initial cracks were due to CB agglomerates, which decreased the mechanical properties of the rubber composites [22,25]. It is evident that distribution of all components in the rubber matrix has an important influence on the fatigue resistance of the composites. In addition, it is not difficult to imagine that if the rubber composite was not well vulcanized, long molecular chains will not form a good cross-linking network, leading to the poor mechanical properties and fatigue life [26].

Based on the fatigue crack propagation theory, if we obtained the crack precursor size, then the fatigue life of the rubber composite is available. Besides, we can quickly judge the mixing effect and stability of the same batch of rubber by the crack precursor sizes, so as to adjust the production process in time, which is significant for guiding the actual production process. Therefore, a reliable calculation method for determining the size of crack precursors is very meaningful. Factors that affect the fatigue life of rubber had been extensively studied [27], but there were few reports on the systematic characterization and analysis of the crack precursor sizes of rubber composites. Crack precursor is an important factor in the fatigue failure of rubber. In our previous work [22], we obtained crack precursor size by computer simulation. However, this method was not easy for laboratory technician to implement, so we needed to find a simple and fast way to get the crack precursor size.

In this study, we introduced three different methods to characterize the crack precursors sizes. The first method, based on the fracture mechanics theory, through simple tension, nicked angle tear, planar tear, and trouser tear experiments, we obtained the crack precursors with sizes of 499.3 μm, 383.6 μm, and 182.3 μm, respectively. The second method, based on the Thomas model [15,22], through crack growth rate test and uniaxial tensile fatigue life test, we obtained the average crack precursor size of 3.3 μm. The third method, through observing the low-temperature brittle fracture morphology of the rubber composites by scanning electron microscopy (SEM), we found that the crack precursor sizes were normally distributed.

## 2. Experimental

### 2.1. Materials

Ethylene propylene diene monomer rubber (EPDM 4045) was supplied by the Petro China Jilin Petrochemical Co., Ltd (Jilin, China); ethylene content was 53.0–59.0 wt% and Mooney viscosity was 38–52 (ML (1 + 4) @100 °C). Carbon black was supplied by Cabot Co., Ltd (Tianjin, China). Other materials used were all commercial reagents. The specific details of the formula were not shown here due to commercial protection.

### 2.2. Materials Preparation

The EPDM composites were prepared by traditional mixed technology. First, the EPDM rubber was mixed with carbon black and other agents in an open mixer. When the mixture was uniformly mixed, a sheet was extruded. Then, the vulcanization characteristics were analyzed with a rotorless vulcanizer (MR-C3, Beijing Ruida Yuchen Co., Ltd, Beijing, China). Finally, the EPDM compound was compression-molded and crosslinked at 165 °C and the pressure of 15 MPa to obtain rubber composite.

### 2.3. Characterization

#### 2.3.1. Nicked Angle Tear Test

**Figure 1:**
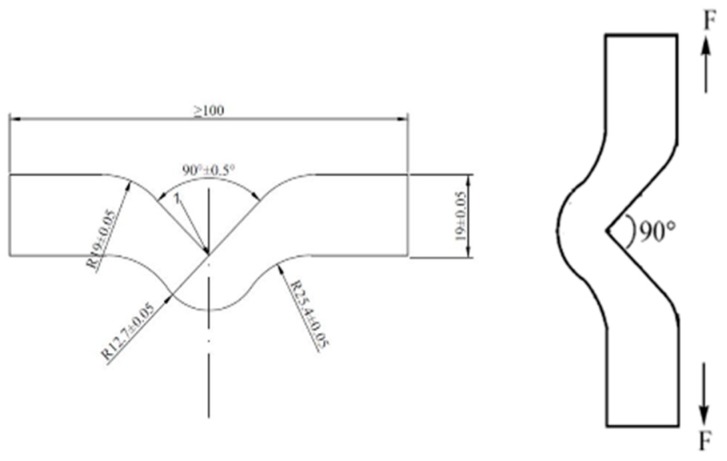
Nicked angle tear test specimen (length 100 mm, width 19 mm, thickness 2 mm, pre-cut angle 90°).

Nicked angle tear sample was shown in Figure 1. The nicked angle tear energy was measured by an electronic tensile machine (CMT4104, Xin Sansi Co., Ltd, Shenzhen, China) according to the ISO 34-1: 2010, the test rate was 500 mm/min.

#### 2.3.2. Planar Tear Test

**Figure 2:**
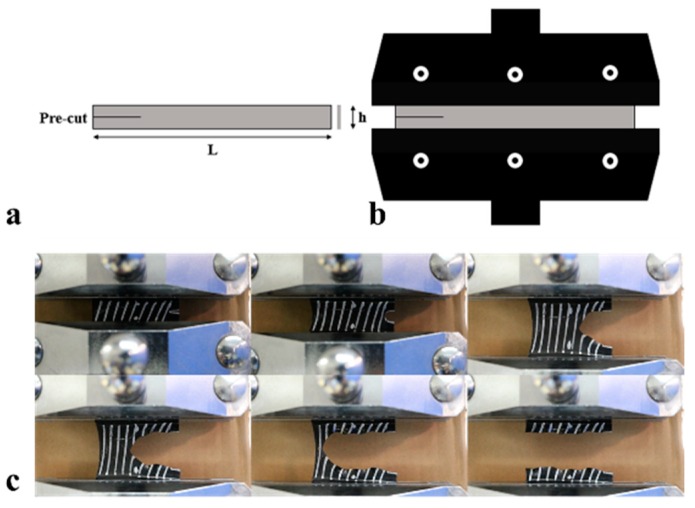
(**a**) Planar tension test specimen (length 100 mm, width 10 mm, thickness 1 mm, pre-cut 15 mm), (**b**) specimen fixture (**c**), shape and location of crack tip at different loading displacements.

The planar tension specimen with pre-cut was shown in Figure 2a. The width (L) was 100 mm, the height (h) was 10 mm, the thickness was around 1 mm, and the pre-cut length was 15 mm. The tear energy was measured by an electronic tensile machine (CMT4104, Xin Sansi Co., Ltd, Shenzhen, China) with the tensile rate 10 mm/min, the fixture was shown in Figure 2b. The stretching process was recorded with a camera as shown in Figure 2c.

#### 2.3.3. Trouser Tear Test

**Figure 3:**
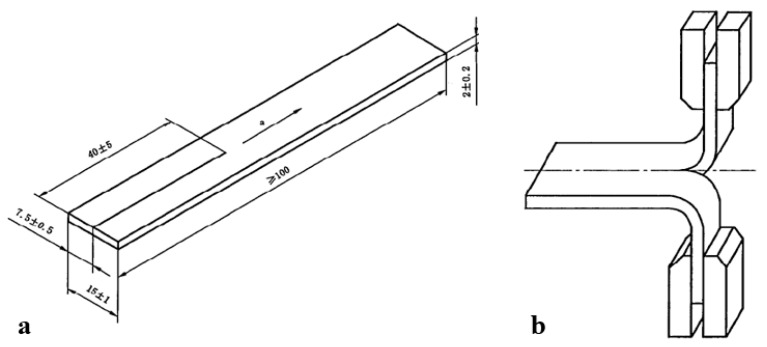
(**a**) Trouser tear test specimen during unloading stage (length 100 mm, width 15 mm, thickness 2 mm, pre-cut 40 mm), and (**b**) the trouser tear specimen during loading stage.

According to the ISO 34-1: 2010, the trouser tear energy was measured by an electronic tensile machine with the tensile rate 100 mm/min. The trouser tear specimen and the tear process during the test were shown in Figure 3a and b, respectively. 

#### 2.3.4. Simple Tension Test

The mechanical properties were measured by an electronic tensile machine (CMT4104, Xin Sansi Co., Ltd, Shenzhen, China) according to the ISO 37: 2011.

#### 2.3.5. Crack Propagation Test

**Figure 4:**
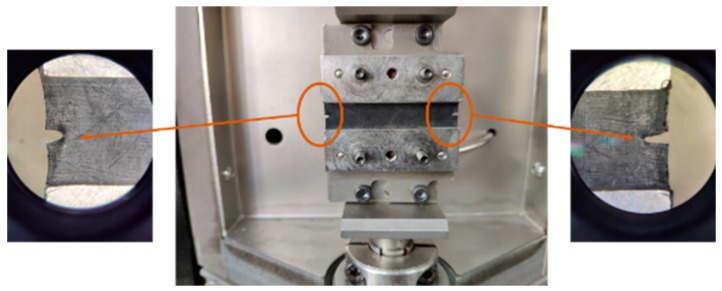
The crack tip and fatigue crack growth test specimen (length 40 mm, width 6 mm, thickness 2 mm, pre-cut 1.5 mm).

As was shown in Figure 4, the crack propagation rates of the rubber composites were determined by a crack extension analyzer (DMA+1000, METRA VIB, France) at a frequency of 20 Hz at 23 °C. The sample size was 2 mm × 6 mm × 40 mm and the pre-cut depth was 1.5 mm [28].

#### 2.3.6. Fatigue Life Test

The fatigue life [29,30] of rubber composites under the condition of maximum strain 150% were obtained through fatigue testing machine (FT3000-2, Beijing Ruida Yuchen Co., Ltd, Beijing, China), according to ISO 6943:2007.

#### 2.3.7. Scanning Electron Microscope

First, the rubber composites were broken into two parts in liquid nitrogen, and then we observed the fracture surface by scanning electron microscope (S-4800, Hitachi Co., Ltd, Japan). Finally, we measured and counted the crack precursor sizes.

## 3. Results and Discussion

### 3.1. Method 1: Critical Tear Energy Method

#### 3.1.1. Theoretical Basis

The specimen’s tear energy under uniaxial stretching can be calculated by the following formula:

(1) $$T=\frac{2\pi Wc}{\sqrt{1+\varepsilon }}$$

When the sample breaks:

(2) $$T_{b}=\frac{2\pi W_{b}c_{0}}{\sqrt{1+\varepsilon_{b}}}$$

where *T* was the tear energy, *T_b_* was the tear energy at break, *W_b_* was the strain energy density, *c*_0_ was the crack precursor size, ε*_b_* was the elongation at break, and *σ_b_* was the tensile strength. The strain energy density *W_b_* was the energy stored per unit volume, which can be quickly estimated:

(3) $$W_{b}\approx \frac{1}{2}\sigma_{b}\varepsilon_{b}$$

So

(4) $$T_{b}=\frac{\pi \sigma_{b}\varepsilon_{b}c_{0}}{\sqrt{1+\varepsilon_{b}}}$$

(5) $$c_{0}=\frac{T_{b}\sqrt{1+\varepsilon_{b}}}{\pi \sigma_{b}\varepsilon_{b}}$$

Suppose

(6) $$T_{b}=T_{c}$$

where *T_c_* was the critical tear energy, then the crack precursor size:

(7) $$c_{0}\approx \frac{T_{c}\sqrt{1+\varepsilon_{b}}}{\pi \sigma_{b}\varepsilon_{b}}$$

#### 3.1.2. Tear Energy Test

##### Nicked Angle Tear

**Figure 5:**
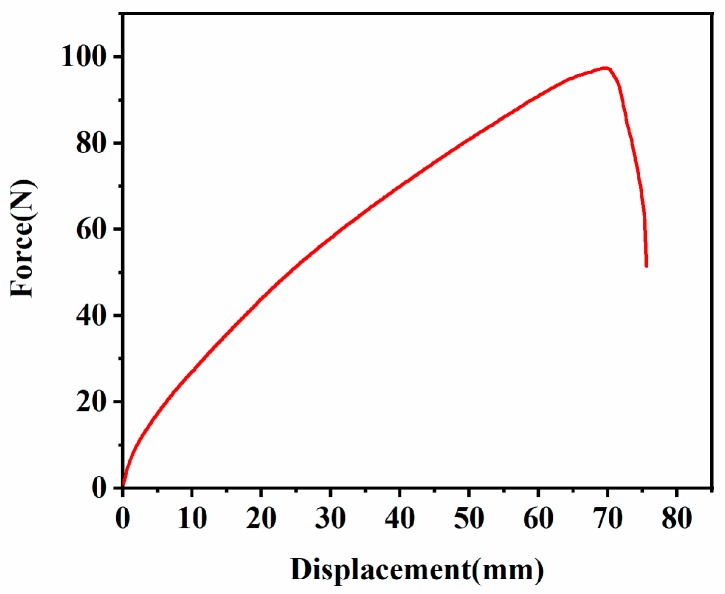
Force-displacement curve of the ethylene propylene diene monomer (EPDM) rubber composite under nicked angle tear test.

The force-displacement curve of nicked angle tear was shown in Figure 5. The force perpendicular to the plane of the cut was applied to the specimen, and the tear strength was calculated as follows:

(8) $$T_{s}=\frac{F}{d}$$

where *T_s_* (kN/m) was the tear strength, *F* (N) was the maximum force required for the sample to tear, *d* (mm) was the thickness of the sample. Because kN/m = kJ/m^2^, *T_s_* and *T_c_* were numerically consistent, and the critical tear energy of the nicked angle tear: *T_c_* = 42.4 kJ/m^2^.

##### Planar Tear

**Figure 6:**
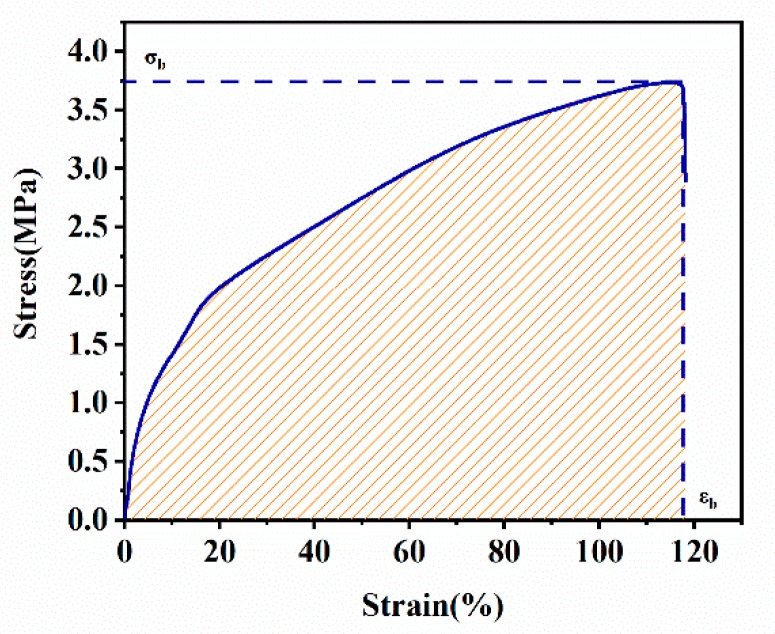
Stress-strain curve of the EPDM rubber composite under planar tension.

The stress–strain curve of the plane tear was shown in Figure 6. Where *ε_b_* was the strain at break, and *σ_b_* was the stress at break. The strain energy density *W* = 3.28 × 10^6^ J/m^3^ was obtained by integrating the stress–strain curve. The sample height *h* was 10 mm, and the critical tear energy of planar tear specimen was calculated by the following:

(9) $$T_{c}=W\times h$$

where *T_c_* = 32.8 kJ/m^2^. 

##### Trouser Tear

**Figure 7:**
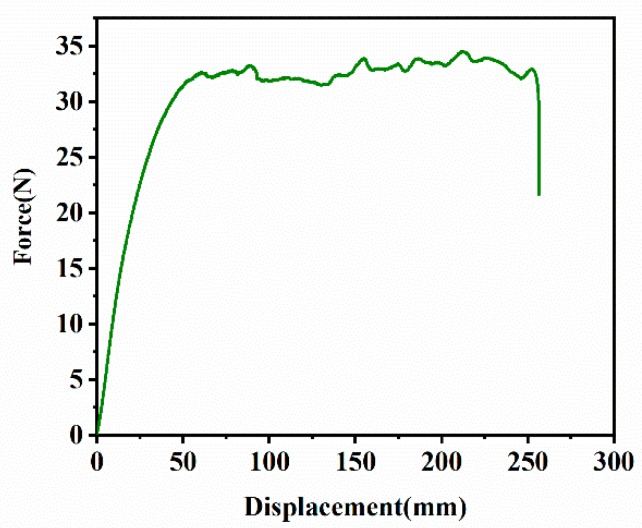
Force-displacement curve of the EPDM rubber composite under trouser tear.

The force-displacement curve of the trouser tear was shown in Figure 7. The maximum force *F* was calculated according to ISO 6133:1998, and the tear strength was calculated according to formula (8), *T_s_* = *T_c_*, so the critical tear energy of trouser tear sample: *T_c_* = 15.6 kJ/m^2^.

#### 3.1.3. Simple Tension Test

**Figure 8:**
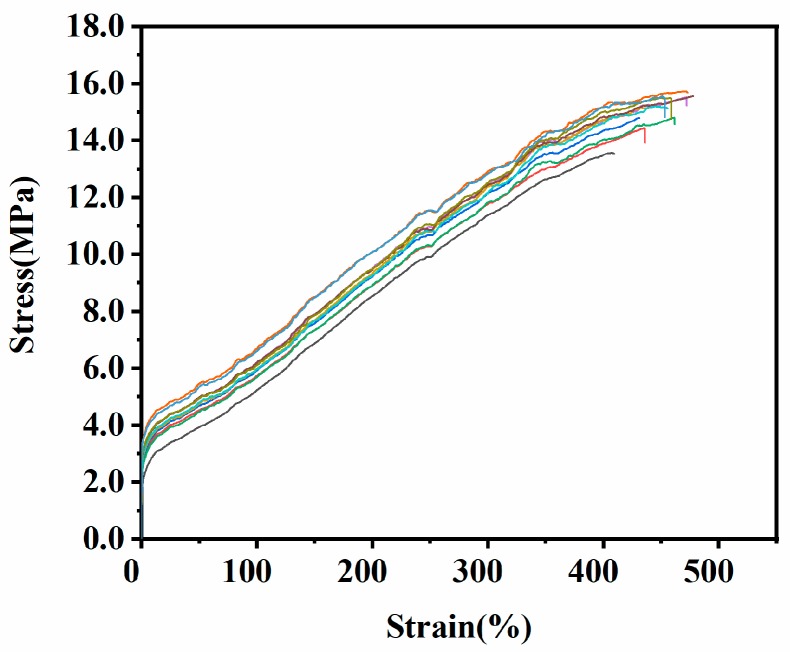
Stress–strain curves of the EPDM rubber composite under simple tension.

The stress–strain curves of simple tension are shown in Figure 8, and the tensile strength and elongation at break of each sample are shown in Table 1. 

**Figure 9:**
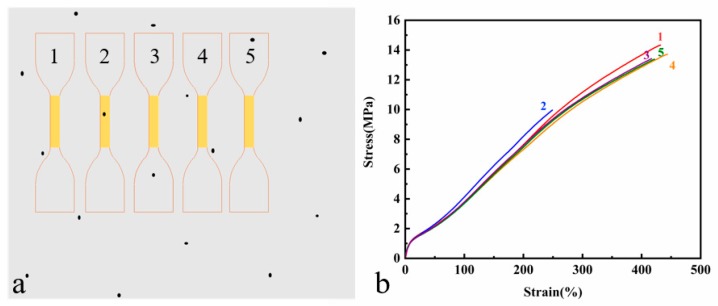
(**a**) Schematic diagram of the distribution of crack precursors on vulcanized rubber sheet (**b**) Stress–strain curves of the five samples under simple tension.

It can be clearly observed that the tensile strength and elongation at break of the same batch of samples were different, which were related to the distribution and size of the crack precursor in the sample. As shown in Figure 9a, crack precursors (black spots) were randomly distributed in the specimen. If the crack precursor was in the yellow working area of the sample, stress concentration would occur at the crack precursor position during the stretching process, thereby the crack precursor further expanding into larger-sized micro-crack, and finally leading to early fracture failure of the specimen, showing less elongation at break and tensile strength shown by the second tensile curve in Figure 9b. The other four samples without crack precursor in work area showed similar elongation at break and tensile strength. Therefore, more than three samples were needed in the test to reduce the error caused by the crack precursor distribution.

#### 3.1.4. Analysis of Crack Precursor Size

**Figure 10:**
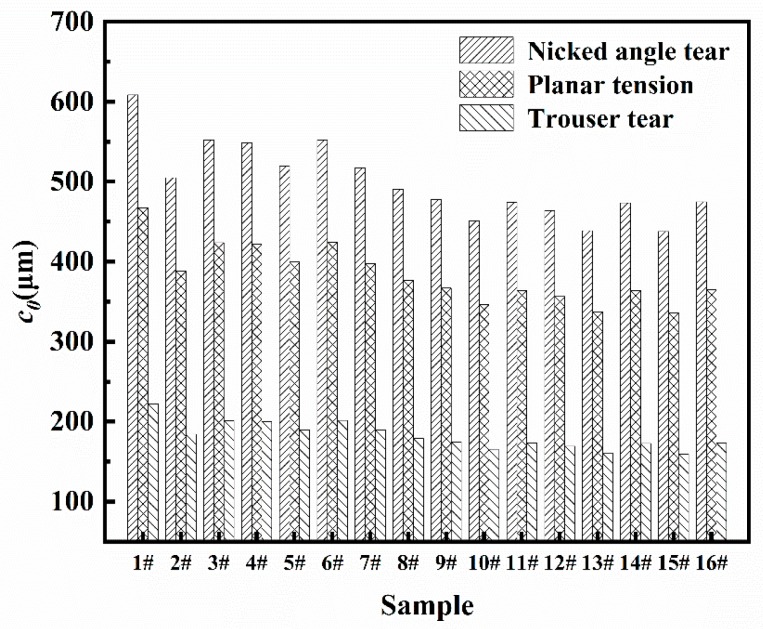
Crack precursor sizes of the different samples under different tear modes.

**Figure 11:**
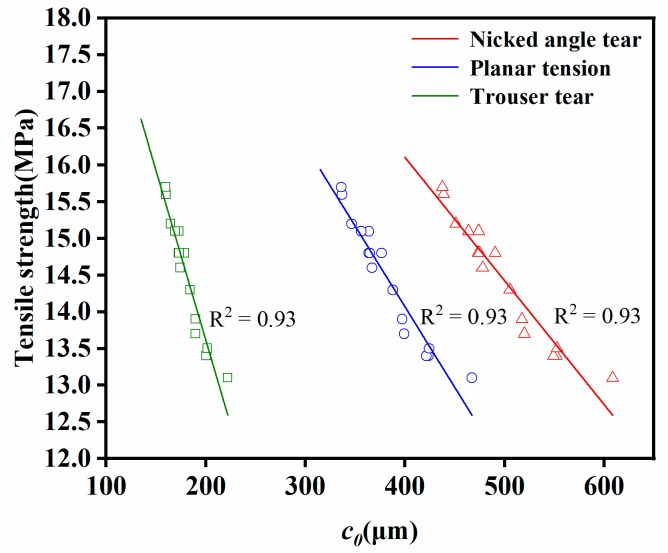
Linear fitting curves of tensile strength and crack precursor size under different tear modes for the EPDM rubber composite.

The crack precursor sizes of samples can be obtained by substituting tensile strength, elongation at break, and critical tear energy under three different modes (nicked angle tear, planar tear, and trouser tear) into formula (7). The crack precursor sizes in different modes were shown in Table 2 and Figure 10. It can be clearly seen that even in the same mode, the crack precursor sizes were different from each other due to the differences in tensile strength and elongation at break of the samples. The tear energy of the same sample in the three modes was also different from each other, therefore, the crack precursor sizes calculated according to the tear energy also had a big difference. In the nicked angle tear mode, the average size of the crack precursor was 499.3 μm. In the planar tear mode, the average size of the crack precursor was 383.6 μm. In the trouser tear mode, the average size of the crack precursor was 182.3 μm. In simple tension tests, the tensile strength and elongation at break were critical and directly related to the error in the calculation results. As shown in Figure 10, the more samples tested for the same sample, the smaller the result error. The linear fitting result of tensile strength and crack precursor size was shown in Figure 11, where the crack precursor size was negatively correlated with tensile strength (R^2^ > 0.9). The larger the crack precursor size, the lower the tensile strength. 

### 3.2. Method 2: Crack Propagation Method

#### 3.2.1. Theoretical Basis

Based on the Thomas fatigue crack growth model [31]:

(10) $$T_{\max}=2\pi W_{\max}c$$

(11) $$r=r_{c}{\left(\frac{T_{\max}}{T_{c}}\right)}^{F}$$

(12) $$\begin{array}{c} N_{f}=\int_{c_{0}}^{\propto }\frac{1}{r}dc=\int_{c_{0}}^{\propto }\frac{1}{r_{c}{\left(\frac{T_{\max}}{T_{c}}\right)}^{F}}dc\\ =\frac{T_{c}^{F}}{\left(F-1\right)r_{c}{\left(2\pi W_{\max}\right)}^{F}}c_{0}^{1-F} \end{array}$$

(13) $$c_{0}={\left(\frac{N_{f}\left(F-1\right)r_{c}{\left(2\pi W_{\max}\right)}^{F}}{T_{c}^{F}}\right)}^{\frac{1}{1-F}}$$

where *c*_0_ is the crack precursor size, *N_f_* is the fatigue life, *F* is the power law index, *r_c_* is the critical crack growth rate, *W*_max_ is the strain energy density [32], and *T_c_* is the critical tear energy measured by the planar tensile test.

#### 3.2.2. Crack Growth Rate Test

The crack growth rates of rubber composites under different tearing energies were determined by crack propagation test platform (DMA+1000). First, the Mullins effect was eliminated with 2000 cycles. Then, the crack growth rates were obtained by observing the distance of the crack tip propagated after a certain number of cycles by a Leica camera [28,33]. The results were shown in Table 3.

**Figure 12:**
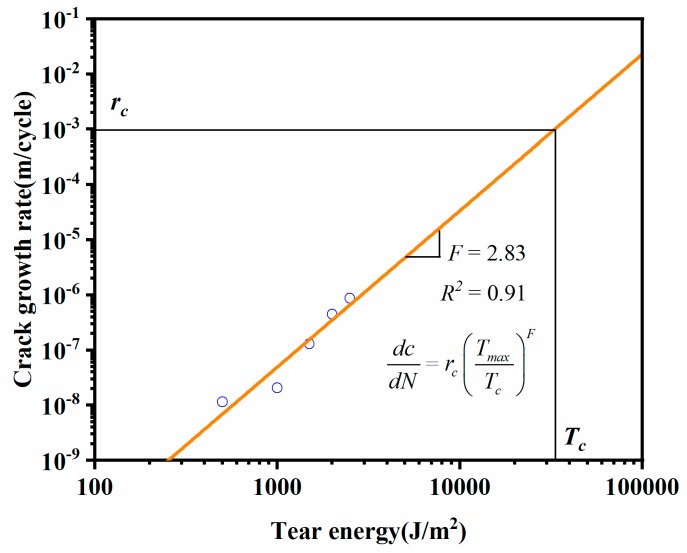
Fitting curve of crack growth rate and tear energy.

The linear fitting result of tear energy and crack growth rate was shown in Figure 12. The critical tear energy was *T_c_* = 3.28 × 10^4^ J/m^2^, corresponding to the critical crack growth rate *r_c_* = 9.73 × 10^−4^ m/cycle. The slope of the fitted line was *F* = 2.83.

#### 3.2.3. Analysis of Crack Precursor Size

In Figure 8, the curve with a strain range of 0–150% was integrated to obtain an average strain energy density *W*_max_ = 4.61 × 10^6^ J/m^3^. The uniaxial tensile fatigue lives of samples at a maximum strain of 150% was shown in Table 4. It can be seen that the fatigue lives of different samples were quite different. So, more samples should be tested as much as possible in the experiment to reduce the error. The above parameters were substituted into the formula (13) to obtain the crack precursor sizes of different samples. As shown in Table 4, the average size of the crack precursor was 3.3 μm. The maximum size of the crack precursor was 2.8 μm smaller than the minimum size, but the fatigue life was reduced by 38,827 cycles. It can be seen that the size of the crack precursor had a direct impact on the durability of the rubber composite.

**Figure 13:**
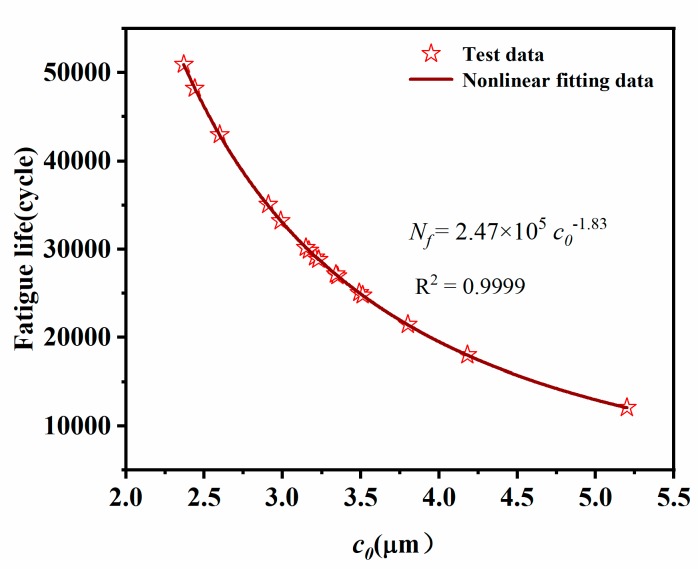
Fitting curve of fatigue life and crack precursor size.

The nonlinear fitting result of crack precursor size and fatigue life was shown in Figure 13. It can be seen that the larger crack precursor size, the shorter the fatigue life of the rubber composite. This was because, during the cyclic loading process, the stress concentration of the crack precursor in the larger size of the rubber composite was more obvious. So that the crack precursors rapidly developed into micro-cracks, which eventually led to the fracture failure of the rubber composites. In Figure 13, the fitting result was very good (R^2^ > 0.999), which can be used to predict the fatigue life of rubber composites.

### 3.3. Method 3: Direct Observation Method by SEM 

**Figure 14:**
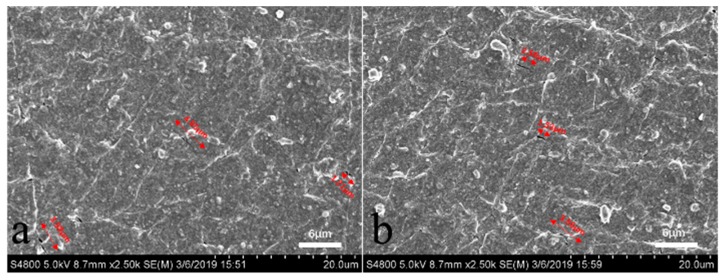
The representative SEM images of EPDM rubber composite surface in different area (**a**) and (**b**).

**Figure 15:**
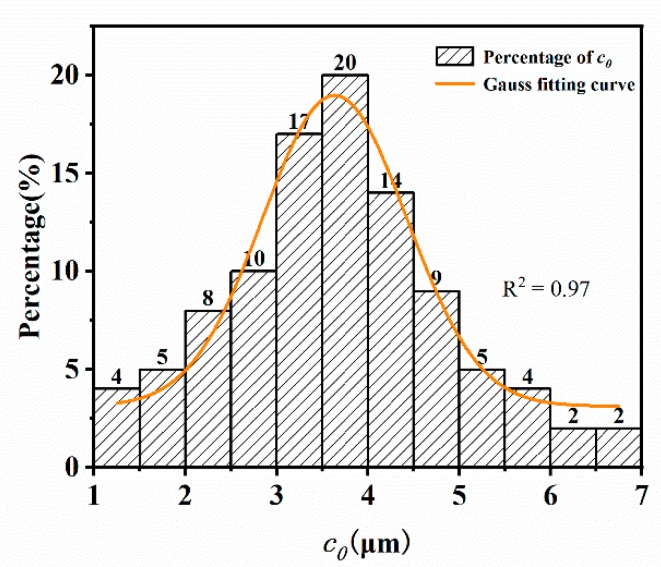
Distributions of the crack precursor sizes.

The cross-sectional morphology of the rubber composite was shown in Figure 14. It can be seen that there were many crack precursors marked by red arrows. These crack precursors were caused by the interfacial separation of zinc oxide particles or carbon black agglomeration particles [27]. We calculated the crack precursor sizes in 10 different SEM photographs. The result showed that the average size of the crack precursor was 3.6 μm. The Gaussian function fitting result was shown in Figure 15, the crack precursor size showed normal distribution, where the correlation coefficient R^2^ = 0.97. It showed that for the same batch of rubber, its crack precursor sizes were distributed in a certain range, and the average size of the crack precursors was closely related to the process.

## 4. Conclusions

(1) The crack precursor size had a great influence on the physical and mechanical properties and fatigue durability of rubber composites. In this study, we characterized and analyzed the crack precursor size of rubber composites by three different methods. 

(2) The first method is critical tear energy method. Based on the theory of fracture mechanics, the average size of the crack precursors obtained by the simple tension test and the critical tear energy in three tear modes were 499.3 μm, 383.6 μm, and 182.3 μm, respectively. The second method is crack propagation method. Based on the Thomas fatigue crack growth model, the average size of the crack precursor was 3.3 μm calculated by the fatigue life and crack growth rate test data of the rubber specimens. The third method is direct observation method by SEM. Observing the cross-section of the rubber composite by SEM, we found that the average size of the crack precursor was 3.6 μm and the crack precursor sizes exhibited a normal distribution. 

(3) By using the first method, we can quickly obtain the sizes of the crack precursors, which can be used for rapid detection of rubber compounding stability. The second method can accurately reflect the sizes of the rubber crack precursors which can be used for further study of fatigue theory. By using the third method, we can directly observe the size and distribution of the real crack precursor and verify the accuracy of the first two methods, but the operation was complicated and time-consuming. Through the above three methods, we systematically characterized and analyzed the crack precursor sizes, which provided theoretical guidance for the in-depth study of the fatigue properties of rubber composites.

## Figures and Tables

**Table 1:** Mechanical properties of different samples.

Samples	Tensile Strength at Failure /MPa	Elongation at Break/%	Stress at 100%/MPa	Stress at 300%/MPa
1#	13.1	370	4.1	11.4
2#	14.3	432	3.7	11.2
3#	13.4	417	3.7	10.7
4#	13.4	422	3.8	10.8
5#	13.7	444	3.7	10.5
6#	13.5	409	5.2	11.4
7#	13.9	436	5.7	11.8
8#	14.8	431	5.9	12.2
9#	14.6	462	5.7	11.8
10#	15.2	472	6.2	12.5
11#	15.1	439	6.0	12.4
12#	15.1	456	6.0	12.2
13#	15.6	478	6.2	12.5
14#	14.8	459	6.1	12.5
15#	15.7	473	6.7	12.9
16#	14.8	453	6.6	12.8

**Table 2:** Crack precursor size *c*_0_ (μm) for different samples.

Sample	*c*_0_ (Nicked Angle Tear)	*c*_0_ (Planar Tension)	*c*_0_ (Trouser Tear)
1#	608.8	467.7	222.2
2#	505.2	388.1	184.4
3#	551.9	423.9	201.4
4#	549.2	421.9	200.5
5#	520.1	399.6	189.8
6#	552.7	424.6	201.7
7#	517.6	397.6	189.9
8#	490.5	376. 8	179.0
9#	477.9	367.2	174.4
10#	451.4	346.8	164.8
11#	474.2	364.3	173.1
12#	464.1	356.5	169.4
13#	438.9	337.2	160.2
14#	473.8	363.9	172.9
15#	437.9	336.4	159.8
16#	475.1	365.0	173.4
average	499.3	383.6	182.3

**Table 3:** Crack growth rates for different tear energy.

Tear Energy (J/m^2^)	Crack Growth Rate (m/cycle)
500	1.15 × 10^−8^
1000	2.06 × 10^−8^
1500	1.30 × 10^−7^
2000	4.47 × 10^−7^
2500	8.72 × 10^−7^

**Table 4:** Fatigue lives (*N_f_*) and crack precursor sizes for different samples.

Sample	*N_f_*	*c*_0_ (μm)
1#	26,978	3.4
2#	29,098	3.2
3#	27,179	3.3
4#	50,921	2.4
5#	21,472	3.8
6#	48,201	2.4
7#	29,881	3.2
8#	30,198	3.2
9#	35,084	2.9
10#	25,064	3.5
11#	24,792	3.5
12#	12,094	5.2
13#	18,025	4.2
14#	33,219	3.0
15#	28,843	3.2
16#	43,005	2.6
